# Differences in disease characteristics and outcomes as determined by biological sex in a large UK idiopathic pulmonary fibrosis population: analysis from the British Thoracic Society, Interstitial Lung Disease registry data

**DOI:** 10.1136/bmjresp-2025-003301

**Published:** 2025-09-25

**Authors:** Sarah Mulholland, Giles Dixon, Matthew Wells, Sam Harding, Paul White, Anne-Marie Russell, Shaney L Barratt

**Affiliations:** 1 Bristol Interstitial Lung Disease Service, North Bristol NHS Trust, Bristol, UK; 2 Academic Respiratory Unit, University of Bristol Faculty of Health Sciences, Bristol, UK; 3 Department of Clinical and Biomedical Sciences, University of Exeter, Exeter, England, UK; 4 Research and Innovation, NHS North Bristol NHS Trust, Bristol, England, UK; 5 Mathematics and Statistics Research Group, University of the West of England, Bristol, UK; 6 School of Medicine and Health, College of Medical and Dental Sciences (MDS) University of Birmingham, Birmingham, West Midlands, UK; 7 University Hospitals Birmingham NHS Foundation Trust, Birmingham, UK

**Keywords:** Idiopathic Pulmonary Fibrosis, Interstitial Fibrosis

## Abstract

**Introduction:**

Growing evidence suggests that biological sex influences the incidence, presentation, diagnosis and outcomes of many lung diseases. Understanding these differences is the first step towards precision medicine to improve patient care.

**Methods:**

In this cross-sectional study, idiopathic pulmonary fibrosis (IPF) patients enrolled in a national (UK), multicentre registry were categorised by sex and analysed for differences in demographics, pulmonary function tests, high resolution CT radiological pattern, eligibility/uptake of antifibrotics and survival.

**Results:**

Of 7177 cases, 77.8% (n=5587) were male, median age 75 years (IQR 69.5–80.5) for both sexes (p=0.83). Males were more likely to have a history of smoking (males 72.9% vs females 60.5%, p<0.001) and lower baseline median forced vital capacity (FVC) % predicted (males 76.4%, IQR 66.2–86.7 vs females 78.8%, IQR 68.6–89.1, p<0.001). Diabetes (males 22.8% vs females 15.1%) and cardiovascular disease (males 58.9% vs females 47.8%) were statistically more common in males (p<0.001), while gastro-oesophageal reflux disease (males 20% vs females 24.6%) and major depressive illness (males 0.8% vs females 2.5%) were more common in females (p<0.001). Significantly, more females experienced symptoms for >24 months prior to first clinic appointment (females 40.1% vs males 36.6%, p=0.03). While more males in the cohort met eligibility criteria for antifibrotics at baseline (pirfenidone FVC 50%–80% males 54.7% vs females 47.6%, nintedanib FVC 50%–80% males 47.0% vs females 41.5%, p<0.001), a larger proportion chose not to commence antifibrotic treatment (males 47.0% vs females 29.6%, p<0.001). Female sex was associated with longer survival; for females, the 75% Kaplan-Meier survival quartile is 7.6 years (95% CI 5.51 to 9.68 years) versus 4.3 years (95% CI 3.82 to 4.78) for males (p<0.001). Male sex (HR 1.76 (95% CI 1.22 to 2.54), p=0.002), higher age (HR 1.042 (95% CI 1.02 to 1.06), p<0.001), lower baseline FVC % predicted (HR 0.98 (95% CI 0.97 to 0.98), p<0.001) and coexistent lung cancer (HR 9.3 (95% CI 2.86 to 30.24), p<0.001) were all independently associated with worse survival.

**Conclusion:**

This is the first UK study to use national registry data to systematically evaluate IPF disease characteristics stratifying by biological sex and highlights distinct characteristics between groups. Future clinical trials should explicitly explore sex-specific targeted interventions and analyses to optimise future IPF patient care.

**WHAT IS ALREADY KNOWN ABOUT THIS TOPIC?**

**WHAT THIS STUDY ADDS?**

- This study is the first to report on UK real-world registry data set to systematically evaluate sex differences in clinical disease characteristics, treatments and outcomes. The results of this study suggest that IPF has distinct characteristics depending on biological sex.

**HOW THIS STUDY MIGHT AFFECT RESEARCH, PRACTICE OR POLICY?**

- These findings highlight key areas that could be addressed to improve patient outcomes and care within the UK. It creates the opportunity to explore tailored approaches to care.

## Introduction

 Pulmonary fibrosis (PF) describes a group of lung diseases where scar tissue progressively and irreversibly replaces normal lung tissue.^1^ There are established sex differences in the incidence and prevalence of various subtypes of PF.^2 3^ Idiopathic pulmonary fibrosis (IPF), the archetypal progressive phenotype, is twice as common in males than females.^1 4^ Lived experience of PF differs between males and females. Independent of age, disease severity or functional capacity, females have greater mental health impairment but fewer physical health-related quality of life (HRQoL) impairments than males.^2^ The underlying reasons for these variances are not known, but small cohorts have implicated differences in environmental exposures^5^ and sex hormones^6^ as potential contributing factors. Understanding these differences is the first step towards precision medicine for patients based on biological sex, to improve patient care.

Antifibrotic medications are known to have similar efficacy and adverse event profiles in males and females.^7 8^ Females are significantly under-represented in these clinical trials. Limited observational registry data currently available has shown males have a shorter time to treatment initiation than females,^9^ which cannot be explained by disease severity alone. The impact of physician–patient sex concordance and implicit bias as a factor contributing to healthcare disparities remains unknown in PF.

Systematic evaluation of sex differences in clinical disease characteristics, treatments and outcomes is urgently needed. We characterised a large UK IPF population by biological sex, determining differences in disease characteristics and outcomes using the British Thoracic Society (BTS) Interstitial Lung Disease (ILD) registry data from January 2013 to October 2024. Baseline characteristics and trends in management of the registry IPF cohort between 2013–2019 and 2013–2023 have been previously published,^10 11^ but did not include detailed exploration of sex differences.

## Methods

### Registry platform and data management

The UK ILD registry has ethical approval (National Research Ethics Service (NRES) reference 12/EE/0381 and renewal 12/EE/0346) to enrol all patients with a definite or probable IPF diagnosis, in accordance with the American Thoracic Society, European Respiratory Society, Japanese Respiratory Society and Latin American Thoracic Society guidelines (ATS/ERS/JRS/LATS),^12^ providing written consent is obtained. The UK IPF Registry is funded by the BTS.

Data are collated and entered by the hospital clinical team responsible for the patient’s care, into a secure web-based platform (https://www.brit-thoracic.org.uk/quality-improvement/lung-disease-registries/bts-ild-registry/) that encrypts identifiable data at the point of entry. Data validation occurs at the point of data entry and includes limiting responses within specific data ranges.^12^ Data can be submitted either prospectively or retrospectively (provided first clinic visit was on or after 1 January 2013), at baseline and then at least every 12 months.

### Patient and public involvement

Patient and public representatives were involved in the original development of the registry programme and have ongoing representation through patient charities. Patients and the public were not directly involved in the study design or conduct of this particular study.

### Antifibrotic use within the UK

In England, antifibrotic medications are prescribed by ILD specialist centres, commissioned by the National Health Service (NHS). Antifibrotics are prescribed by general hospitals in Scotland, Wales and Northern Ireland, where commissioning differs. Pirfenidone has been provided through the NHS since 2013 (originally technology appraisal 282 (TA282),^13^ with nintedanib approved in 2016 (TA379).^14^ The original technology appraisal guidance for IPF enabled the prescription of pirfenidone or nintedanib for patients diagnosed with IPF who have a forced vital capacity (FVC) of 50%–80% predicted. Recommendations were amended in 2023, additionally authorising the use of nintedanib in patients with an FVC>80% predicted (TA864).^15^ Baseline FVC% predicted became the accepted marker of disease progression to determine the eligibility of patients for antifibrotics.

### Project methodology

A data access request was submitted to the BTS-ILD registry (September 2023) and approved by the BTS-ILD registry committee in February 2024. Access to the complete data set from 1 January 2013 (date of origin) to the censor date of 15 October 2024 was obtained in October 2024. Patients with a documented diagnosis of IPF and recorded biological sex were included in the analysis.

Data collated included age, sex, ethnicity, smoking history, duration of symptoms, comorbidities, presenting radiological pattern of PF on high-resolution computed tomography (HRCT) scan (definite usual interstitial pneumonia (UIP), probable UIP or indeterminate for UIP),^12^ lung function tests (forced expiratory volume in 1 s (FEV1), FVC, transfer factor of the lung for carbon monoxide (TLCO) and transfer coefficient of the lung for carbon monoxide (KCO)), 6 min walk tests and Medical Research Council (MRC) dyspnoea scale, treatments, management and outcomes. GAP (gender, age, physiology) stage was calculated using the available data.^16^

### Data analysis

Data were analysed using Statistical Package for the Social Sciences (International Business Machines corporation, Chicago, USA, V.29.0.2.0). Descriptive statistics were used to characterise baseline demographic and clinical characteristics of the patient cohort according to biological sex. In addition, differences were sought between the two sexes on antifibrotic eligibility at baseline (using baseline FVC % predicted as per NHS guidance), the uptake of antifibrotics by patients and the holistic care of patients in terms of oxygen assessment and referral for pulmonary rehabilitation, palliative care or lung transplantation.

Data three SD above or below the median were independently sense checked and excluded from the analysis of that variable if deemed erroneous.

Continuous data were expressed as median with IQR and compared using the Mann Whitney U test. Categorical data were expressed as number and percentage and compared using χ^2^ test of association.

Kaplan-Meier survival curves were constructed to compare biological sex groups and analysed using the Log-rank test; a p-value of less than 0.05 was considered statistically significant. Mortality risk factors were explored using Cox-regression analysis among the predefined variables of sex, smoking history, baseline FVC and comorbidities. Interaction terms were tested to determine whether the effect of covariates differed by sex. Kaplan-Meier survival curves were constructed and Cox-regression analysis undertaken in a subgroup of the cohort with complete data sets and registered up until and including October 2019 allowing follow-up of at least 5 years.

## Results

### Participating hospitals

At the time of data censorship, 64 hospitals were actively participating in the registry (online supplemental appendix 1); 13 were specialist ILD centres in England, 40 were secondary care centres in England and 11 were centres across the devolved nations (Scotland, Wales and Northern Ireland, where commissioning differs).

### Baseline characteristics

Between 1 January 2013 and 15 October 2024, n=7177 cases of IPF were enrolled into the UK IPF registry with data available.

Baseline demographics are detailed in table 1. Most patients were male 77.8% (n=5587/7177), with a median age 75 years (IQR 69.5–80.5) for both sexes (p>0.05). Where ethnicity was recorded, White British predominated in both males (n=846, 93.6%) and females (n=254, 92.4%). In February 2023, the question wording changed within the ILD registry to explicitly separate sex and gender questions. Our study is purely concerned with the analysis of sex differences.

**Table 1:** Baseline demographics and characteristics of UK idiopathic pulmonary fibrosis (IPF) cohort between January 2013 and October 2024, stratified by biological sex

	Number of patients with data	Male	Female	P value
Biological sex (n, %)	7177	5587 (77.8%)	1590 (22.0%)	**<0.001**
Age (median, IQR)	7102	75 (69.5–80.5)	75 (69.5–80.5)	0.83
Smoking status (n, %)	5619			
Ever smoker		3229 (72.9%)	718 (60.5%)	**<0.001**
Never smoker		1203 (27.1%)	469 (39.5%)	
Ethnicity (n, %)	1179			
White (British)		846 (93.6%)	254 (92.4%)	0.48
Other		58 (6.4%)	21 (7.6%)	
Comorbidities (n, %)*				
Cardiovascular disease	5227	2434 (58.9%)	522 (47.8%)	**<0.001**
Diabetes	5227	941 (22.8%)	169 (15.5%)	**<0.001**
Gastro-oesophageal reflux disease	5227	825 (20.0%)	269 (24.6%)	**<0.001**
Major depressive illness	5227	34 (0.8%)	27 (2.5%)	**<0.001**
Lung carcinoma	5227	42 (1.0%)	14 (1.3%)	0.45
Other malignancy	5227	87 (2.1%)	16 (1.5%)	0.18
BMI>30	3302	938 (33.1%)	285 (35.8%)	0.16
Pulmonary hypertension or right heart strain (n,%)	907	150 (21.4%)	49 (23.9%)	0.44
Baseline lung function (median, IQR)				
FEV1% predicted	5736	81.4 (70.5–92.3)	80.5 (67.5–93.5)	**<0.001**
FVC% predicted	5741	76.4 (69.5–80.5)	78.8 (68.6–89.1)	**<0.001**
TLCO% predicted	4012	49.5 (38.7–60.3)	49.2 (38.9–59.6)	0.44
KCO% predicted	3837	79.2 (64.8–93.7)	77.4 (65.5–89.4)	0.18
MRC dyspnoea at diagnosis (n, %)	1422			
1–2		567 (51.2%)	154 (49.0%)	0.50
3–4		523 (47.2%)	151 (48.0%)	0.78
5		18 (1.6%)	9 (2.9%)	0.16
GAP stage at diagnosis (n, %)	3801			
I (points 0–3)		1015 (33.3%)	532 (70.9%)	**<0.001**
II (points 4–5)		1742 (57.1%)	217 (28.9%)	**<0.001**
III (points 6–8)		294 (9.6%)	1 (0.1%)	**<0.001**

Statistically significant values highlighted in bold.

* Comorbidities are not mutually exclusive.

%, percentage; BMI, Body Mass Index; FEV1, forced expiratory volume in 1 s; FVC, forced vital capacity; GAP, gender age physiology; KCO, coefficient of the lung for carbon monoxide; m, metres; MRC, Medical Research Council, dyspnoea scale; n, number; p value, statistical probability value; TLCO, transfer factor of the lung for carbon monoxide.

Males were more likely to have a history of smoking (males n=3229/4432, 72.9% vs females n=718/1187, 60.5%, p<0.001) and have a lower median FVC % predicted at presentation (males 76.4%, IQR 69.5–80.5 vs females 78.8%, IQR 68.6–89.1, p<0.001). There was no statistical difference in the baseline TLCO and KCO based on biological sex (p>0.05).

The list of comorbidities recorded in the registry is not exhaustive (table 1). Diabetes and cardiovascular disease were statistically more common in males (p<0.001), while gastro-oesophageal reflux disease and major depressive illness were more common in females (p<0.001).

Females were more likely to present with a lower GAP stage compared with males; approximately 70% females had baseline GAP stage I compared with only one-third of males (GAP stage 1: males n=1015, 33.3% vs females n=532, 70.9%, p<0.001), although MRC dyspnoea scores did not differ between groups.

### Referral symptoms and diagnosis

At the point of enrolment into the Registry, the majority of patients had experienced symptoms of exertional breathlessness and/or cough for >12 months (males n=2584/4246, 60.9% vs females n=736/1171, 62.9%), although significantly more females had experienced symptoms for >24 months (females 40.1% vs males 36.6%, p=0.028) (table 2).

**Table 2:** Symptoms and diagnosis of patients with idiopathic pulmonary fibrosis (IPF), stratified by biological sex

	Number of patients with data	Male	Female	P value
Duration of symptoms prior to first appointment	5417			
No symptoms		85 (2.0%)	21 (1.8%)	0.65
Symptoms<6 months		614 (14.5%)	150 (12.8%)	0.15
Symptoms 6–12 months		963 (22.7%)	264 (22.5%)	0.92
Symptoms 12–24 months		1029 (24.2%)	266 (22.7%)	0.28
Symptoms>24 months		1555 (36.6%)	470 (40.1%)	0.03
Diagnosis by consensus MDT (n, %)	6019			
Discussed at presentation		4394 (93.1%)	1200 (92.4%)	0.155
Radiological pattern (n, %)	5117			
Definite UIP		1726 (42.8%)	463 (42.6%)	0.87
Probable UIP		2081 (51.7%)	576 (52.9%)	0.45
Indeterminate		124 (3.1%)	27 (2.5%)	0.30
Alternative to UIP		98 (2.4%)	22 (2.0%)	0.43
Surgical biopsy (n,%)	5074			
Performed		193 (4.9%)	72 (6.6%)	0.02
Not performed		3774 (95.1%)	1034 (93.4%)	
Histological diagnosis (n, %)				
Definite UIP		114 (2.9%)	50 (4.5%)	0.06
Probable UIP		69 (1.7%)	19 (1.75%)	0.96
Indeterminate for UIP		10 (0.3%)	4 (0.4%)	0.54

Statistically significant values highlighted in bold.

%, percentage; MDT, multidisciplinary team; n, number; p value, statistical probability value; UIP, usual interstitial pneumonia.

Most patients in the cohort were discussed in a multidisciplinary team meeting (MDT) to reach a consensus diagnosis (males 93.1% and females 92.4%), with no significant difference between the groups (table 2). The presenting radiological pattern of fibrosis on HRCT did not differ statistically between biological sexes, with 94.5% (n=3807/4029) males and 95.5% (n=1039/1088) females having reported definite or probable patterns of UIP (p>0.05).

While the need for a video-assisted thoracoscopic lung biopsy to reach a consensus diagnosis was small across the entire cohort (n=265/5074, 5.2%), more females required a lung biopsy to reach a consensus than did males (females n=72, 6.6%, males n=193, 4.9%, p=0.022, table 2).

### Management and outcomes

Table 3 provides details on the management of IPF within the registry and correlates with the UK NICE (National Institute for Health and Care Clinical Excellence) quality standards.^17^ Taking the year of presentation into consideration and the baseline FVC % predicted, more males met the FVC NICE eligibility criteria for antifibrotic therapy than females (pirfenidone: males 54.7% vs females 47.6%, p<0.001 or nintedanib: males 47.0% vs females 41.5%, p<0.001). A larger proportion of males chose not to start antifibrotic treatment even if they were eligible (males n=284/604, 47.0% vs females n=118/398, 29.6%, p<0.001). More females were documented within the registry as not meeting eligibility for antifibrotics, despite fulfilling NICE criteria (females n=157/398, 39.4% vs males n=66/604, 10.9%, p<0.001). The reasons for this are not clear. Missing data did not allow comparison of antifibrotic side effect profiles, reasons for stopping antifibrotics or dose reduction between groups.

**Table 3:** Antifibrotic treatment and management of idiopathic pulmonary fibrosis (IPF) patients at enrolment to registry, stratified by biological sex

	Number of patients with data	Male	Female	P value
Eligible for antifibrotic based on FVC (n, %)*	5964			
Pirfenidone (FVC 50%–80%)		2555 (54.7%)	616 (47.6%)	**<0.001**
Nintedanib (FVC 50%–80%)		2196 (47.0%)	537 (41.5%)	**<0.001**
Nintedanib (FVC>80%)		431 (9.2%)	129 (10%)	0.41
Antifibrotic prescribed within 3 months (n,%)*	5910			
Nintedanib		1365 (29.5%)	352 (27.6%)	0.19
Pirfenidone		1025 (22.1%)	215 (16.8%)	**<0.001**
Neither		2255 (48.7%)	716 (56.1%)	**<0.001**
Reason for not prescribing antifibrotic in eligible patients (n, %)	1002			
Patient choice		284 (47.0%)	118 (29.6%)	**<0.001**
Caution/contraindication		18 (3.0%)	14 (2.5%)	0.64
Does not meet criteria		66 (10.9%)	157 (39.4%)	**<0.001**
Other†		232 (38.4%)	109 (27.4%)	–
Hospital unable to prescribe		4 (0.4%)	0 (0.0%)	0.10
Immunosuppression in previous 3 months (n, %)	5224	261 (6.4%)	96 (8.5%)	0.02
Assessed for oxygen therapy	3456			
Assessment at first visit		2396 (88.2%)	635 (85.9%)	0.16
Referred for pulmonary rehabilitation	4090			
Assessed		2685 (88.9%)	769 (88.6%)	0.79
Referred		1726 (53.6%)	453 (52.2%)	0.47
Not referred		1496 (46.4%)	415 (47.8%)	0.47
Course completed<12 months ago		180 (5.6%)	53 (6.1%)	0.56
Patient declined		242 (7.5%)	78 (9.0%)	0.15
Unsuitable		717 (22.3%)	185 (21.3%)	0.55
Referred for lung transplantation (n,%)	4776			
Yes		72 (1.9%)	21 (2.0%)	0.86
Not applicable currently		1271 (34%)	378 (36.2%)	0.19
Not applicable ever		2390 (64%)	644 (61.7%)	0.18
Palliative care needs assessed	4344	2691 (77.1%)	744 (79%)	0.21

Statistically significant values highlighted in bold.

* Subgroups of pirfenidone and nintedanib not mutually exclusive.

† Category not further defined in registry information.

%, percentage; FVC, forced vital capacity; n, number; p value, statistical probability value.

Although not a recommended treatment for IPF (NICE Clinical Guidance (CG) 163),^18^ females were statistically more likely to have received immunosuppressive medications during their treatment pathway (females 8.5% vs males 6.4%, p=0.02). There was no significant difference in percentages of patients assessed for oxygen, pulmonary rehabilitation or lung transplantation, based on biological sex (table 3).

**Figure 1:**
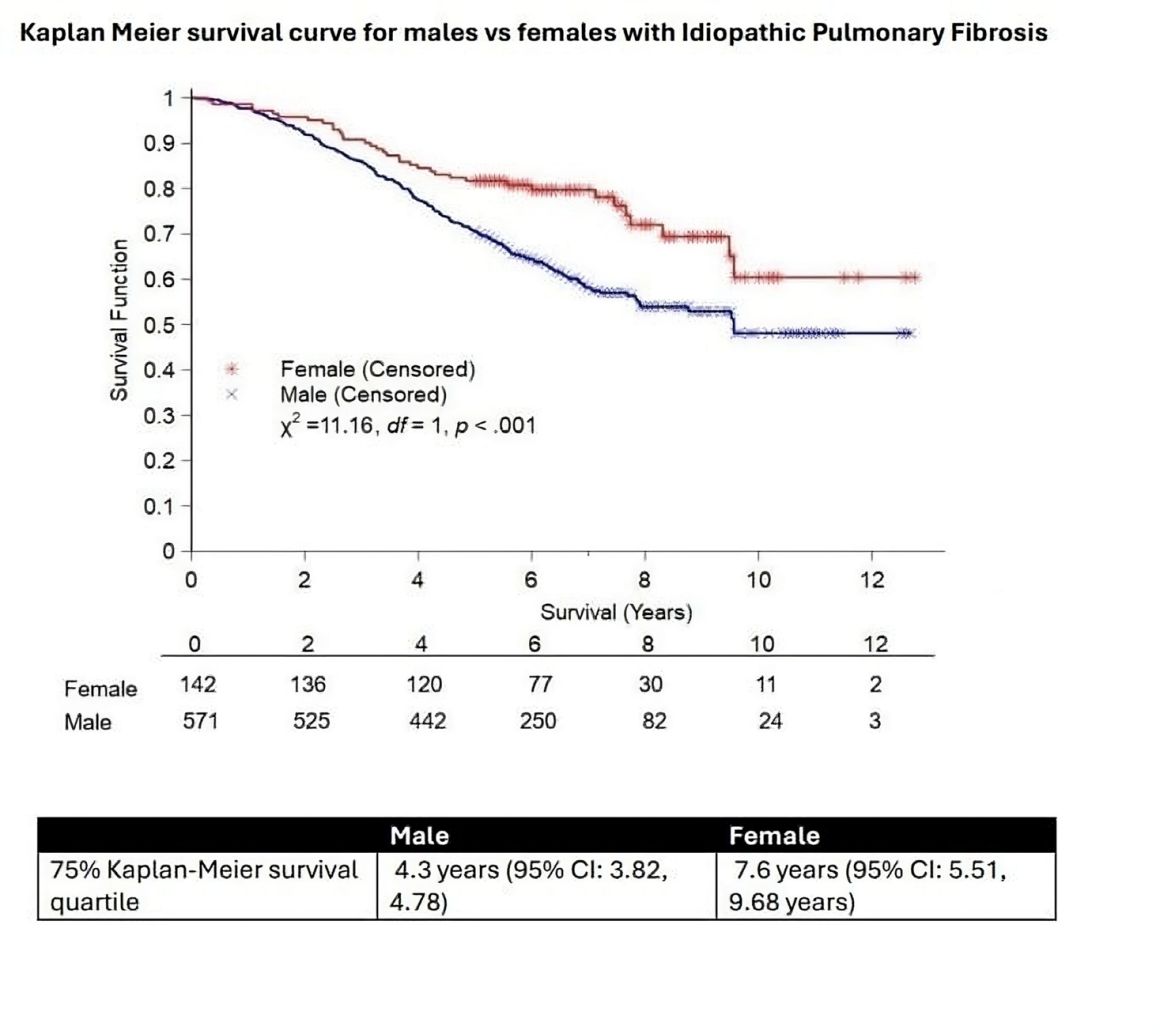
Kaplan-Meier survival curve for idiopathic pulmonary fibrosis (IPF) patients stratified by biological sex with table of patients at risk. Female sex was associated with longer survival (female 75% Kaplan-Meier survival quartile 7.6 years (95% CI 5.51 to 9.68 years)) vs males 4.3 years (95% CI 3.82 to 4.78), p<0.001). Median survival for males was estimated to be 9.6 years and median survival time for females was not achieved.

**Figure 2:**
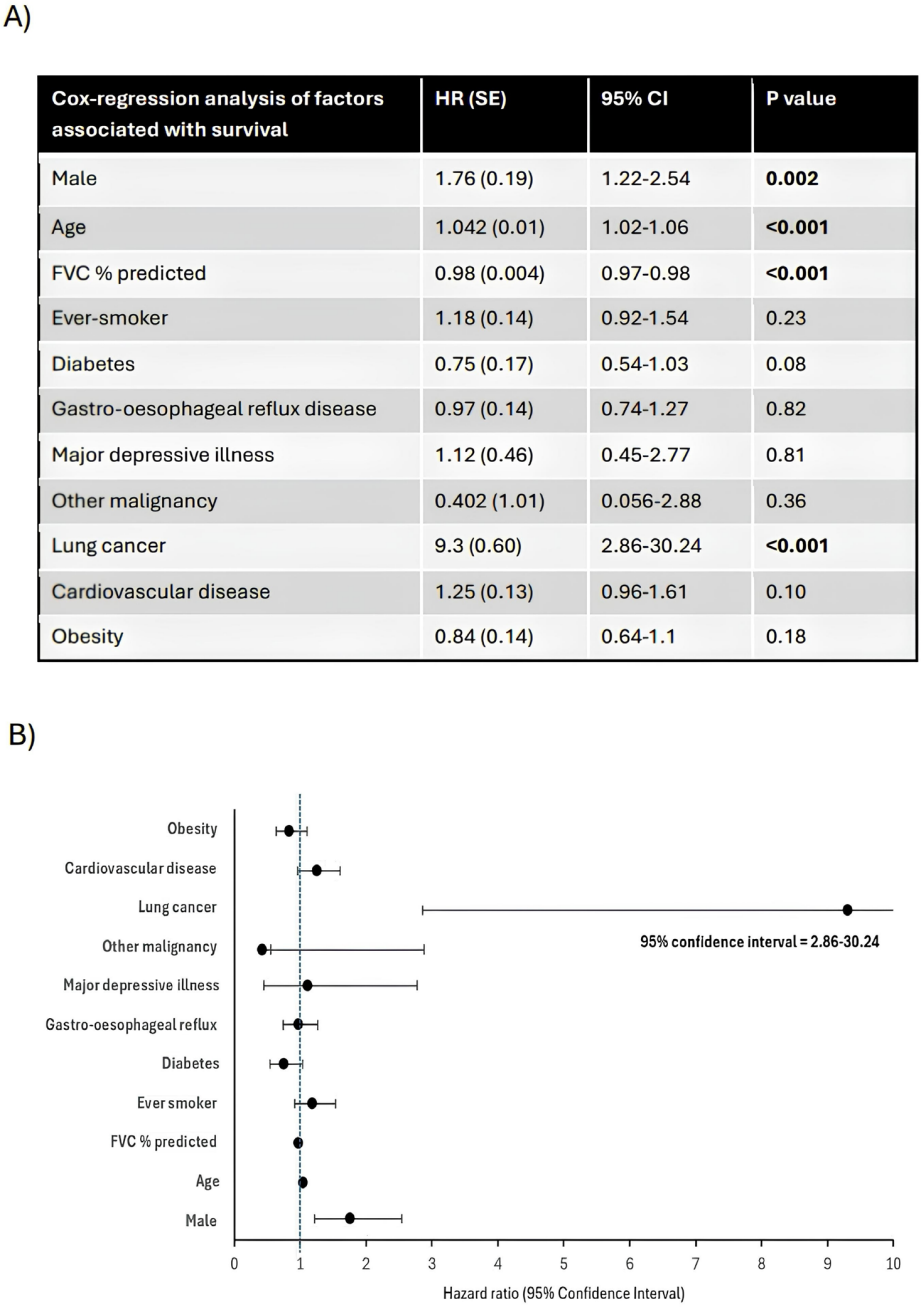
**A**) Cox-regression analysis of factors independently associated with survival. Statistically significant values highlighted in bold. P value, statistical probability value; %, percentage. (B) Forest plot of HRs and 95% CIs for associations in Cox-regression.

In a cohort of 713 patients with complete data sets for FVC % predicted, age, sex, smoking and comorbidities registered up to and including October 2019 (allowing a 5 year follow-up period), female sex was associated with longer survival (p<0.001). For females, the 75% Kaplan-Meier survival quartile was estimated to be years (95% CI 5.51 to 9.68 years), and for males, the corresponding 75% quartile was estimated to be 4.3 years (95% CI 3.82 to 4.78). Median survival for males was estimated to be 9.6 years and median survival time for females was not achieved (figure 1). Cox-regression was also performed on this cohort. Male sex, higher age, lower baseline FVC % predicted and coexistent lung cancer were all independently associated with worse survival (figure 2). The strongest independent association with IPF survival was coexistent lung cancer (HR 9.3, 95% CI 2.86 to 30.24, p<0.001) while male sex was associated with HR of 1.76 (95% CI 1.22 to 2.54, p<0.002). Interaction terms indicated that there was no significant difference in the effect of age, FVC, smoking status or comorbidities on survival between sexes (p>0.05, data not shown).

## Discussion

This is the first UK study to use BTS-ILD registry data to evaluate the baseline characteristics, treatment, management and outcomes of patients with IPF, stratifying by biological sex. The results of this study suggest that IPF has distinct characteristics depending on biological sex with notable differences in baseline disease severity, comorbidities, duration of symptoms prior to referral, need for lung biopsy to make a consensus MDT diagnosis and overall survival. The study highlights key areas that should be explored to understand potential reasons underlying these sex differences in IPF, to facilitate tailored person-based care and to improve patient outcomes.

In this cohort, males presented with more severe disease, having a lower FVC % predicted and higher GAP stage than females at baseline. This is in keeping with the findings of a multicentre prospective French IPF cohort (n=236, COhorte FIbrose, COFI),^5^ who found that females tended to present with less advanced disease at diagnosis, better lung function and more preserved FVC. The authors hypothesised that this might be due to lower exposure to triggering agents such as tobacco smoke or occupational inhalants compared with men, although recognised future changes in lifestyle habits may influence this. Reflecting on the differing smoking history exposure in our own cohort, our findings could support this hypothesis.

Barriers to timely diagnosis in ILD are well described^19^ and the UK BTS ILD registry data have previously highlighted the negative impact that even 6–12 months delay in assessment can have on survival in IPF.^20^ The data from this cohort are no exception. Patients experienced a significant length of time between the onset of their symptoms and clinical assessment. Furthermore, more females had symptoms for >24 months at their first clinic visit compared with males. While some of these observations may be related to workforce-related challenges and waiting times, it may also relate to differences in lived experience of the disease. Han *et al* demonstrated that independent of age, disease severity or functional capacity, females have greater mental health impairment but fewer physical HRQoL impairments than males.^2^ The associations between self-efficacy, social support and sex pattern in PF are unknown. Gendered expectations may be an influencing factor and worthy of further exploration.

Sex differences were identified in the frequency of the dominant comorbidities. Although the list recorded in the registry was not exhaustive, cardiovascular disease and diabetes were more common in males, while gastro-oesophageal reflux disease and major depressive illness were more common in females. This is in keeping with a study of 348 Swedish IPF registry participants, where coronary artery disease and other cardiovascular diseases, such as atrial fibrillation and heart failure, were more prevalent in males, but thyroid disease and osteoporosis were more common in females.^3^ While shared risk factors, for example, smoking history, may explain this finding, Izbicki *et al*^21^ found that the association between IPF and increased risk of developing coronary artery disease was independent of these common confounding factors, but a mechanistic explanation for this observed association was not clearly defined.

Previous studies have indicated that definite or probable UIP patterns of fibrosis on HRCT are more commonly found in male patients, facilitating an IPF diagnosis. Conversely, atypical radiological patterns are more frequent in female patients, necessitating a surgical lung biopsy to confirm the diagnosis of IPF.^5 22^ In this cohort, we found that while there was no significant difference in the proportions of HRCTs demonstrating definite or probable UIP patterns, more females required a lung biopsy to secure an MDT diagnosis of IPF. Assayag *et al* have previously investigated the role of gender in making a confident diagnosis of IPF and concluded that there is evidence for bias when using sex to inform an ILD diagnosis, which may result in men being overdiagnosed and women underdiagnosed with IPF.^22 23^ The finding that statistically more females were prescribed immunosuppressive medications, not a currently recommended treatment strategy for IPF (CG163), may suggest a degree of unconscious bias towards non-IPF diagnoses of these patients during their pathway.

The data suggest that while males were more likely to meet NICE eligibility criteria for antifibrotics at baseline, there was a significant proportion of males that declined to take antifibrotics through patient choice, and this was significantly higher than in females (males 47.0% vs females 29.6%). Other real-world data sets have suggested that both sex and ethnicity may affect uptake of antifibrotic medications, with the potential for subsequent impact on patient outcomes. A study of n=14 792 US veterans with IPF showed that while uptake of antifibrotics was generally low in this cohort (17% overall), there were significant disparities in adoption, with lower uptake associated with female sex (adjusted OR, 0.41; 95% CI, 0.27 to 0.63; p<0.001), Black race (adjusted OR, 0.60; 95% CI, 0.49 to 0.73; p<0.001) and rural residence (adjusted OR, 0.88; 95% CI, 0.80 to 0.97; p=0.012).^24^ Similarly, Ghimire *et al* 2024 demonstrated racial and sex disparities in antifibrotic prescriptions in a large US cohort of IPF patients (n=10 667).^25^ Although disease prevalence was relatively even among sexes (46.6% males vs 53.3% females), there was a significantly higher prescription of antifibrotics among Caucasians and males (76.6% males vs 39.4% females), with improved hospital outcomes for those on antifibrotic therapy (information on lung function testing was not provided).

In this cohort, there were insufficient complete data sets available to compare antifibrotic side effect profiles, reasons for stopping antifibrotics or dose reduction between groups. Adherence to antifibrotic treatment in IPF was recently studied in an Italian prospective cohort. Among 667 new IPF cases, 296 received antifibrotic prescriptions (77% male), with 62.8% being adherent in the first year. While the number of female patients receiving antifibrotics was lower, adherence was not significantly associated with sex or age, comorbidity score, the number of concomitant drugs or antifibrotic used.^26^ Conversely, Ortiz *et al*^27^ showed in their multicentre Spanish cohort of 232 IPF patients commencing antifibrotics that while time to first adverse event and all-cause mortality were similar between the antifibrotic medications, females were more likely to withdraw from therapy due to side effects (predominantly gastrointestinal). Female sex, diarrhoea and photosensitivity were independent factors associated with an increased risk for definitive discontinuation from therapy. Furthermore, posthoc analyses of clinical trial data have shown more frequent dose reductions, treatment interruptions and early discontinuations of antifibrotic medications in females.^28^ Given that females were significantly under-represented in the clinical trials of antifibrotics, additional real-world studies are required to explore these factors in more detail.

In this UK cohort, females demonstrated a significantly better survival outcome compared with males, with worse survival associated independently with male gender, increasing age, lower baseline FVC and coexistent lung cancer. This is in keeping with the large body of evidence that contributed to the development of the GAP model used to help predict mortality in IPF.^16^ In a single-centre retrospective study of IPF patients in Italy,^29^ women also demonstrated better survival and better tolerance to long-term therapy compared with males. Interestingly, no gender differences emerged in terms of reduction in/discontinuation of therapy, nor did this impact mortality. There are, however, contrasting reports as to the influence of sex and gender on survival in the broader literature. A multicentre, prospective study of French IPF patients demonstrated no survival differences based on biological sex,^5^ although the authors comment that this may have been due to small sample size and lack of statistical power. The UK parliamentary brief on Men’s Health gives important context to the significant disparities in male life expectancy, between some groups of men within the UK and across a range of diseases.^30^ The reasons underpinning this are likely to be numerous and have interconnected influence; health engagement, health literacy, social determinants and behaviour can all vary with an individual’s culture, community and socioeconomic status. Consequently, several stakeholders and experts have called for a national men’s health strategy in the UK.

As with all real-world data sets, there are limitations. The first limitation concerns that of missing data which we have reflected transparently on within the results section. We acknowledge that the statistical robustness of the survival data and generalisation of the Cox-regression results to the entire cohort may have been biased by both missing data and the immaturity of data in terms of follow-up. The Registry relies on individual contributing centres to provide up-to-date mortality outcomes, therefore potentially biasing the survival analysis. Non-sensical data entry also contributed to missing data items, for example input of oxygen saturation of 0%. A recent update to the registry platform (November 2024) has ensured that it is now impossible to enter data that falls outside strict validation boundaries and should improve the quality of data received. Second, data entry for specialist centres was mandated in 2022 but was voluntary prior to that date and remains so for participating secondary care hospitals. Selection of patients, therefore, is an important source of bias. The paper describes >7000 patient entries from 64 UK hospitals, but we are unable to provide a breakdown of number of cases per hospital to help understand potential bias. Data entry is dependent on local resource allocation, which was significantly impacted during the COVID pandemic, resulting in low data entry during 2020–2021. Mandated data entry for specialist centres and data linkage to national mortality databases will hopefully drive future patient enrolment and ensure robustness of the data but will not fully resolve potential issues surrounding generalisability to all IPF patients seen within secondary care.

## Conclusion

The results of this study suggest that IPF has distinct characteristics depending on biological sex. Additional research is required to explore and understand potential reasons underlying sex differences in IPF to facilitate tailored person-based care and to improve patient outcomes. The recorded data did not allow for analysis by gender. Gender, separate to biological sex, may account for, or contribute to, differences noted in the data and this should be explored so that it can be accounted for, or absented accordingly. In order to achieve this, it is important that future clinical trials explicitly explore sex-specific and gender-specific targeted interventions and undertake detailed gender and sex analyses, taking into account different presentations, environmental risk factors and lived experiences of this population to optimise future IPF patient care.

## Supplementary material

10.1136/bmjresp-2025-003301 online supplemental file 1

## Data Availability

Data may be obtained from a third party and are not publicly available.
