# Supplementary material for: Differences in disease characteristics and outcomes as determined by biological sex in a large UK idiopathic pulmonary fibrosis population: analysis from the British Thoracic Society, Interstitial Lung Disease registry data
Source: BMJ Open Respir Res. 2025 Sep 25;12(1):e003301. doi: 10.1136/bmjresp-2025-003301 (PMC12481391; doi:10.1136/bmjresp-2025-003301)
Supplement: online supplemental file 1 [file bmjresp-12-1-s001.docx]

Appendix 1: List of participating hospitals

**England**

Addenbrooke's Hospital, Cambridge University Hospitals NHS Foundation Trust

Birmingham Heartlands Hospital, Heart of England NHS Trust

Castle Hill Hospital, Hull and East Yorkshire Hospitals NHS Trust

Central Middlesex Hospital, London North-West Healthcare NHS Foundation Trust

Cheltenham General Hospital, Gloucestershire Hospitals NHS Foundation Trust

Churchill Hospital, Oxford University Hospitals NHS Trust

City Hospital, Sandwell and West Birmingham NHS Trust

Countess of Chester Hospital, Countess of Chester Hospital NHS Foundation Trust

Croydon University City Hospital, Croydon Health Services NHS Trust

Darlington Memorial Hospital, County Durham and Darlington NHS Foundation Trust

Ealing Hospital, London North-West Healthcare NHS Foundation Trust

George Eliot Hospital, George Eliot Hospital NHS Trust

Glenfield Hospital, University Hospitals of Leicester

Gloucestershire Royal Hospital, Gloucestershire Hospitals NHS Foundation Trust

Good Hope Hospital, Heart of England NHS Trust Guy’s Hospital,

Guy’s and St Thomas’ NHS Foundation Trust Hammersmith Hospital,

Imperial College Healthcare NHS Trust Harrogate District Hospital,

Harrogate and District NHS Foundation Trust Hinchingbrooke Hospital,

Hinchingbrooke Health Care NHS Trust King's College Hospital,

King’s College Hospital NHS Foundation Trust King’s Mill Hospital,

Sherwood Forest Hospitals NHS Foundation Trust

Liverpool Heart & Chest Hospital, Liverpool Heart & Chest Hospital NHS Foundation Trust

Musgrove Park Hospital, Taunton & Somerset NHS Foundation Trust

New Cross Hospital, Royal Wolverhampton Hospitals NHS Trust

Norfolk and Norwich University Hospital, Norfolk & Norwich University Hospitals NHS Foundation Trust

North Devon District Hospital, Northern Devon Healthcare NHS Trust

Northern General Hospital, Sheffield Teaching Hospitals NHS Foundation Trust

North Middlesex University Hospital NHS Trust

Northwick Park Hospital, London North-West Healthcare NHS Foundation Trust

Nottingham City Hospital, Nottingham University Hospitals NHS Trust

Papworth Hospital, Papworth Hospital NHS Foundation Trust

Peterborough City Hospital, Peterborough & Stamford Hospitals NHS Foundation Trust

Queen Alexandra Hospital, Portsmouth Hospitals NHS Trust

Queen Elizabeth Hospital, Gateshead Health NHS Foundation Trust

Royal Brompton Hospital, Royal Brompton and Harefield NHS Foundation Trust

Royal Derby Hospital, University Hospitals of Derby & Burton NHS Foundation Trust

Royal Devon and Exeter Hospital, Royal Devon & Exeter Foundation NHS Trust

Royal Free Hospital, Royal Free London NHS Foundation Trust

Royal Lancaster Infirmary, University Hospitals of Morecambe Bay NHS Foundation Trust

Royal Victoria Infirmary, Newcastle upon Tyne Hospitals NHS Foundation Trust

Russell’s Hall Hospital, The Dudley Group NHS Foundation Trust

Solihull Hospital, Heart of England NHS Trust

Southampton General Hospital, University Hospital Southampton NHS Foundation Trust

Southmead Hospital, North Bristol NHS Trust

St James' University Hospital, Leeds Teaching Hospital NHS Trust

St Mary's Hospital, Imperial College Healthcare NHS Trust

University College Hospital, University College London Hospitals NHS Foundation Trust

University Hospital, University Hospitals Coventry & Warwickshire NHS Trust

University Hospital Aintree, Aintree University Hospitals NHS Foundation Trust

University Hospital of North Midlands, University Hospitals of North Midlands NHS Trust

University Hospital of North Tees, North Tees & Hartlepool NHS Foundation Trust

Wansbeck Hospital, Northumbria Healthcare NHS Foundation Trust

Worcester Royal Hospital, Worcestershire Acute Hospitals NHS Trust

Wythenshawe Hospital, Manchester University NHS Foundation Trust

**Scotland**

Aberdeen Royal Infirmary, NHS Grampian

Glasgow Royal Infirmary, NHS Greater Glasgow and Clyde

Lorn & Islands District General Hospital, NHS Greater Glasgow and Clyde

Royal Alexandra Hospital, NHS Greater Glasgow and Clyde

Vale of Leven District General Hospital, NHS Greater Glasgow and Clyde

**Wales**

Glan Clwyd Hospital, Betsi Cadwaladr University Health Board

University Hospital Llandough, Cardiff and Vale University Health Board

Wrexham Maelor Hospital, Betsi Cadwaladr University Health Board

**Northern Ireland**

Antrim Area Hospital, Northern Health and Social Care Trust

The Ulster Hospital, South-Eastern Health and Social Care Trust
